# *Pharmacology Research & Perspectives* calls for pharmacology education papers

**DOI:** 10.1002/prp2.772

**Published:** 2021-05-11

**Authors:** Jennifer Martin, Michael Jarvis

**Affiliations:** ^1^ University of Newcastle Newcastle Australia; ^2^ University of Illinois‐Chicago Chicago USA

Since its inception, *Pharmacology Research & Perspectives (PR&P)* has championed the publication of state‐of‐the‐art pharmacological science and practice. Key strategies for the journal have focused on advancing pharmacology research rigor and transparency, highlighting early career researcher science, and publishing reviews and commentaries on topical issues covering all facets of basic and clinical pharmacology. Specific examples include recent *PR&P* virtual issues on pharmacological research methodology, early career researcher studies, negative research findings, and sex as an experimental variable.

In the context of decreasing academic pharmacology departments globally, and the continued integration of pharmacological science into siloed and reductionistic biomedical research specialization, comprehensive and rigorous training in pharmacology is essential. This is key to both the growth of pharmacological science, but perhaps more importantly, the generation of reliable translational knowledge of drug action and the development of new clinically useful pharmacological interventions. Broadly, the *PR&P* portfolio of topical special and virtual issues provides important foundational educational and “best practice” resources. However, basic gaps in the development of critical analysis for the processes that affect humans’ responses to drugs or endogenous molecules, processes due to altered physiology or pathophysiology, predictive statistics, environmental and other relevant variables remain. Additionally, trainee opportunities to gain integrated perspectives of rapidly evolving biomedical subdisciplines, including Bayesian modeling, chemical biology, quantitative systems pharmacology, in silico modeling, pharmacogenomics, and pharmacokinetic/dynamic relationships, are necessarily limited in highly focused grant‐driven research environments.

To further enhance our efforts in fostering the training of scientists and practitioners involved in all aspects of pharmacology, *PR&P* announces the establishment of Pharmacological Education as a new topical section of the journal and a Call for Papers in Pharmacology Education. The *PR&P* Author Guidelines have been revised and detail specific areas of pharmacological education focus covering basic and clinical pharmacology and related biomedical science and practice. All manuscript types (original research, short reports, reviews, and commentaries) are welcome. Papers that describe integrated approaches to the development and critical analysis of pharmacological principles for specialists and related biomedical scientists are encouraged. Readers will be very interested in the paper and associated commentary by Dr. Kelly Quesnelle and colleagues^1^, ^2^in which they report on the development of a novel integrated approach to pharmacology training within a medical curriculum. The *PR&P* Editorial Board is delighted to facilitate scholarly communications in the pharmacology education area and we look forward to an interactive dialog among pharmacology educators, scientists, and clinicians on this important topic.
